# Effect of enzymolytic soybean meal supplementation on performance, nitrogen excretion, serum biochemical parameters and intestinal morphology in broilers fed low-protein diets

**DOI:** 10.5713/ab.23.0091

**Published:** 2023-06-26

**Authors:** Xin Zhu, Kai Gao, Ziyi Zhang, Haiying Liu, Guiqin Yang

**Affiliations:** 1 College of Animal Science and Veterinary Medicine, Shenyang Agricultural University, Shenyang 110866, China

**Keywords:** Chicken, Enzymolytic Soybean Meal, Growth Performance, Intestinal Health, Low Crude Protein Diet, Nitrogen Excretion

## Abstract

**Objective:**

The objective of this study was to investigate the effect of supplementation with enzymolytic soybean meal (ESBM) on broilers fed low crude protein (CP) diets.

**Methods:**

A total of 360 one-day-old broilers were randomly assigned to six treatments with 6 replicates per treatment and 10 chicks per replicate for a period of 42 days. Chicks were fed a basal standard high-CP diet as a positive control (PC), a low-CP diet (reducing 10 g/kg CP from the PC) as a negative control (NC), or an NC + 0.5%, 1.0%, 1.5%, or 2.0% ESBM diet.

**Results:**

Compared to chicks fed the PC, chicks fed the NC had a decreased body weight gain (BWG, p<0.05) from 1 to 42 days, but supplementation with 2.0% ESBM restored BWG (p<0.05) and even linearly improved the feed conversion rate (FCR, p<0.05). Digestibility of CP and ether extract was increased (p<0.05) in chicks fed a 1.0% ESBM diet compared to the PC. With increasing levels of ESBM, nitrogen (N) excretion decreased (p<0.05). The addition of ESBM to the diet did not affect (p>0.05) serum concentrations of total protein, albumin and total cholesterol but led to a descending trend in triglycerides and an ascending trend in calcium and urea N at 42 days (p<0.10). There were no differences (p>0.05) in villus height (VH), crypt depth (CD), and VH/CD (V/C) of the duodenum and jejunum between the PC and NC at both 21 days and 42 days, while increasing dietary ESBM levels linearly (p<0.05) decreased CD and increased V/C of the duodenum and jejunum at both 21 days and 42 days.

**Conclusion:**

The findings indicated that ESBM could be used in broiler low-CP diets to improve production performance, decrease N excretion, and enhance intestinal health.

## INTRODUCTION

Chickens are an important source of animal protein for human beings, and approximately 65 billion chickens are produced annually worldwide [1]. With the population of the world increasing, there is a growing gap between the demand for chicken meat and the supply of broiler production [2]. In the commercial broiler sector, the feeding cost accounts for approximately 70% of the total production cost. Among those feedstuffs, protein is one of the most expensive nutrients in commercial poultry feed, and the level of dietary protein is the main determinant of feed cost in commercial broiler chicken production [3]. Due to the shortage and high price of protein feed, increasing the utilization efficiency of plant protein feed through various feed processing technologies, reducing dietary protein levels and using synthetic amino acids are important strategies for reducing feed costs and controlling nitrogen emissions, which has become a hotspot in poultry feed research in recent years [4,5].

Soybean meal (SBM) is a byproduct of soybean oil processing and is the most widely used protein source in broiler diets. SBM usually has a high protein content (45% to 55%) and a balanced amino acid composition compared with other plant protein sources. However, SBM still contains some anti-nutritional factors, such as soybean antigen proteins and trypsin inhibitor, which are harmful to the growth and intestinal health of broilers [6]. *In vitro* enzymolytic hydrolysis and fermentation of SBM is an effective way to degrade soybean protein, further remove anti-nutritional factors and release soluble protein and small peptides from SBM, thus improving the protein utilization efficiency and bioavailability of the diet [7,8]. This enzymolytic SBM (ESBM) has been shown to reduce anti-nutritional factors and have a higher level of amino acids and more digestible amino acids than SBM, thus improving the protein quality [9–11]. In addition, ESBM has also been found to improve growth performance, immune response, intestinal health and microbiota composition in aquatic and farm animals [12–14].

Lowering dietary crude protein (CP) levels in broiler feed supplemented with feed-grade amino acids has been reported to diminish the broiler sector’s substantial dependence on plant protein feed sources, such as SBM, and attenuate flows of undigested protein into the hindgut to stimulate fermentation by potential pathogens, thus contributing to maintaining intestinal health and decreasing N excretion [15,16]. However, reducing the dietary CP level to a more aggressive extent could permanently depress the production performance of broilers even though all indispensable amino acids were provided [17]. Furthermore, adding excess nonbound synthetic amino acids into the diet could produce an imbalance of digestion dynamics between energy and protein, which can lead to impaired net protein deposition and extravagant N excretion [18]. Thus, we speculated that adding ESBM into a low-CP diet could maintain the performance of broilers comparable to those fed a standard high-CP diet. In this study, therefore, we supplemented ESBM in low-CP diets and evaluated its effect on growth performance, N excretion, serum biochemical parameters, and intestinal morphology in broilers.

## MATERIALS AND METHODS

### Animal care

The present experiment was reviewed and approved by the Animal Care and Use Committee of Shenyang Agricultural University (No. 201906018).

### Experimental design, animals and diets

A total of 360 commercial day-of-hatch, mixed sex Arbor Acres broiler chicks were obtained from a commercial hatchery (Shenyang, China). These chicks were randomly assigned to one of 6 dietary treatments, with each treatment consisting of 6 replicates of 10 chicks per treatment. Chicks fed a basal corn-SBM standard high-CP diet formulated to meet the recommended nutritional levels from Arbor Acres’s guidelines were used as a positive control (PC); a negative control (NC) diet was formulated by reducing 10 g/kg CP content from a PC diet; and experimental diets with varied levels (0.5%, 1.0%, 1.5%, and 2.0%) of ESBM were formulated by replacing SBM with ESBM of an NC diet with constant CP contents. Both SBM and ESBM were supplied by Liaoning Complete Biotechnology Co. Ltd. (Shenyang, Liaoning, China), and the nutrient compositions of SBM and ESBM are shown in Table 1. The biochemical characteristics of SBM and ESBM components are as follows: trichloroacetic acid-soluble protein 1.87% and 18.98%, glycinin 128.95 and 2.58 mg/g, β-conglycinin 95.14 and 4.91 mg/g, and trypsin inhibitor 9.69 mg/g and ND (undetected), respectively. The composition of the experimental diets is provided in Table 2, and the levels of indispensable amino acids in each group were consistent.

The chicks were raised in three-layer wire cages (length 120 cm×width 60 cm×height 30 cm) in an environmentally controlled broiler room. The temperature and environment programs followed the Arbor Acres Broilers Management guide. Chicks were provided *ad libitum* access to water and feed throughout the entire experiment.

### Growth performance and sample collection

All chicks were weighed by replicate in the early morning after a 12-h period of feed deprivation at the beginning (d 1), d 21 and the end (d 42) of the experimental period. Feed consumption was measured per replicate at the same time. These values were used to calculate the average daily feed intake (ADFI), average daily gain (ADG), and feed conversion ratio (FCR). Body weight gain (BWG), feed intake (FI), and mortality were recorded for each replicate.

On the mornings of d 21 and d 42, blood samples (approximately 10 mL) were collected into vacuum blood collection tubes via the jugular vein from 1 bird randomly selected from each replicate. The serum was separated by centrifugation (3,000×g for 10 min at 4°C) and stored at −20°C until analysis. On d 21 and d 42, the same birds (1 bird per replicate) were euthanized by cervical dislocation. Segments of the mid-duodenum and mid-jejunum were removed and rinsed with cold physiological saline (0.9% saline) and then stored in 10% buffered formalin immediately.

### Chemical analysis

Samples of diet, fecal excretion and ESBM were analyzed according to the procedures of the AOAC (2006) for dry matter (DM), nitrogen (N), ether extract (EE), ash, calcium (Ca), and phosphorous (P). Gross energy (GE) was determined using an automatic adiabatic oxygen bomb calorimeter (IKA-C2000, Automatic Energy Analyzer; IKA-Werke Gmbh, Staufen, Germany). Amino acids in the ESBM were assayed using ion-exchange chromatography with an automatic amino acid analyzer (Euro-EA-3000, Automatic Amino Acid Analyzer; EuroVector Technology, Hudson, NH, USA) after hydrolyzing with 6 N HCl at 110°C for 24 h.

### Measurement of serum parameters

Determination of serum total protein, albumin, total cholesterol, triglyceride, calcium, and blood urea nitrogen (BUN) levels was conducted using a spectrophotometer (Ao Yi A360; AOE Instruments, Shanghai, China) following the instructions of the kit’s manufacturer (Nanjing Jiancheng Bioengineering Institute, Nanjing, China).

### Measurement of intestinal morphology

Each intestinal segment sample was embedded in paraffin, cut into 5-μm serial sections, and stained with hematoxylin-eosin for identification. The villus height (VH) and crypt depth (CD) of each section were measured under a light microscope (CK-40; Olympus, Tokyo, Japan) at 40 × magnification and analyzed with an Image Analyzer (Lucia Software; Lucia, ZaDrahou, Czech Republic).

### Statistical analysis

All data were analyzed using one-way analysis of variance in SPSS 19.0 statistical software (Statistical Package for Social Science; SPSS Inc., Chicago, IL, USA). Replicates were considered the experimental units in the data. Means separation was performed through orthogonal contrasts for linear and quadratic effects of incremental additions of ESBM, with polynomials determined by the Interactive Matrix Language procedure of SPSS. Differences between means were compared using least significant difference multiple range tests, and differences were considered significant if p<0.05 and trends if 0.05≤p<0.10.

## RESULTS

### Growth performance

As shown in Table 3, compared with PC, ADG in NC was significantly decreased (p<0.05) from 22 to 42 days and from 1 to 42 days. There was no significant difference (p>0.05) in ADG between the ESBM groups and NC group, but a positive linear trend in ADG with increasing levels of ESBM was observed from 1 to 21 days (p = 0.067) and from 1 to 42 days (p = 0.088). Broilers fed a 2.0% ESBM diet had a comparable (p>0.05) ADG with those in the PC group from 1 to 42 days. There were no significant differences in the ADFI (p>0.05) among all groups, but the FCR decreased linearly (p<0.05) with increasing levels of ESBM from 1 to 21 days, from 22 to 42 days and from 1 to 42 days. In addition, no mortality was observed in any group during the entire experimental period (data not shown).

### Nutrient digestibility

The digestibility of CP and EE was affected (p<0.05) and had a quadratic increasing trend (p<0.10) by supplementation with ESBM (Table 4). The 1.0% ESBM group had the highest (p<0.05) CP and EE digestibility compared with the other groups. The apparent digestibilities of DM, calcium, total phosphorus and GE were not different among all groups (p>0.05).

### N excretion

The results from Table 5 show that total N intake and digested N were greater (p<0.05) in the PC group than in the NC group, although there was no significant difference (p>0.05) in apparent N digestibility between the PC and NC groups. Dietary supplementation with ESBM affected total N intake, digested N and apparent N digestibility (p<0.05). Compared to that in the PC, the N content of excreta in the NC group was significantly decreased (p<0.05). Adding ESBM to the diet significantly affected and linearly reduced (p<0.05) the N content of excreta and N excretion of the diet.

### Serum parameters

There were no significant differences (p>0.05) in serum total protein, albumin, total cholesterol, triglyceride, calcium and urea N at both 21 days and 42 days between the PC and NC groups (Table 6). Linear decreasing trends (p<0.10) of triglyceride, calcium and urea N at 42 days were observed with increasing dietary ESBM levels, while serum concentrations of total protein, albumin and total cholesterol were not significant (p>0.05) at both 21 days and 42 days among ESBM groups.

### Intestinal morphology

As shown in Table 7, no significant differences (p>0.05) in VH, CD and V/C of the duodenum and jejunum at both 21 days and 42 days were found between the PC and NC groups, except that CD in the jejunum of the NC group was greater (p<0.05) than that of the PC group at 42 days. In the duodenum, supplementation with ESBM affected (p<0.05) VH, CD and V/C at 21 days and CD and V/C at 42 days. CD was decreased and V/C was increased at both 21 days and 42 days with the increasing dietary level of ESBM in a linear manner (p<0.05). Similarly, in the jejunum, adding ESBM to the diet affected (p<0.05) CD and V/C at 21 days and VH, CD, and V/C at 42 days. With increasing levels of ESBM, CD decreased and V/C increased at both 21 days and 42 days in a linear manner (p<0.05). Among all groups, the 2.0% ESBM group showed the highest (p<0.05) V/C of the duodenum and jejunum compared with the other groups at both 21 days and 42 days.

## DISCUSSION

The nutritional quality of vegetable protein feedstuff can be improved by biotechnological processing methods, which is conducive to an increased share in the feed market and sustainable development in the feed industry. Along with the development of industrial enzyme production, various stable enzymes with high potency are commercially available and have been used for the treatment of plant protein feed to cleave target chemical bonds and reduce antinutritional factors [19]. Ma et al [10] and Ma et al [13] found that after enzyme treatment, the antigen protein of SEM can be effectively modified, and functional peptides are released, thus posing less of a threat to the gastrointestinal tracts of animals. In this study, over 96% of antigen proteins, including glycinin, β-conglycinin, and trypsin inhibitor, were decreased after compound enzyme treatment, and trichloroacetic acid-soluble proteins were increased from 1.87% to 18.98% in ESBM, indicative of increased nutrition values. Compared to those fed a SEM diet, broilers fed a 2.0% ESBM diet showed a similar ADG during the starter and whole period, and with increasing dietary ESBM levels, FCR linearly decreased throughout the whole period. These results were consistent with the previously published results by Wang et al [20], who reported that replacing SBM with ESBM at 50, 100, and 150 g/kg in the diet linearly increased FI and ADG with increasing levels of ESBM, and FCR showed a linear and quadratic decrease with higher ESBM inclusion, but it did not show any further improvement at ESBM levels higher than 100 g/kg. Similarly, Shen et al [21] fed ESBM to broilers at levels of 0%, 0.3%, 0.6%, and 0.9% and found that the ESBM level increased in the diet and the FCR decreased. Although an improvement in growth performance by dietary ESBM supplementation was observed in this study, a linear increase in ADG did not reach a plateau, and thus, higher levels of ESBM should be included in the diet that will be researched in the future.

Notably, in this study, broilers fed a low-CP diet showed a compromised ADG, but not FI and FCR, compared to those fed a standard high-CP diet, suggesting that feeding a low-CP diet to broilers produced a negative effect on growth performance. A diet formulation strategy that reduces CP content but is simultaneously supplemented with free indispensable and dispensable amino acids based on ideal amino acid profiles of the animal has been put into practice for many years to decrease SBM demand and N excretion. In terms of growth performance, a variety of pictures of the effects of low dietary CP on broilers have been provided to date. For instance, results from Lambert et al [16] and Maynard et al [18] showed that the reduction of dietary CP by no more than 3% points did not compromise BWG, FI, and FCR in broilers, while Brandejs et al [22] found that decreasing dietary CP from 20% to 18% significantly decreased the body weight of broilers, even though a reduced CP diet was supplemented with crystalline amino acids to match the standardized ileal digestible amino acid profiles. One possible explanation for the compromised performance of broilers fed a low-CP diet observed in this study could be the varied fate of free amino acids vs. protein-bound amino acids and the worse profiles of ideal amino acids, even though the main indispensable amino acids, such as lysine, methionine and threonine, were supplied in a low-CP diet. Interestingly, inclusion of 2.0% ESBM in a low-CP diet could maintain the growth performance of broilers compared to those fed a standard high-CP diet in this study. A possible reason may be that more small peptides and indispensable amino acids, which were crucial to improve the efficiency of growth in juvenile animals [23], were available in the soluble part of ESBM, as observed in that the trichloroacetic acid-soluble protein reached 18.98% in this study.

In this study, we found that dietary inclusion of 0.5% and 1.0% ESBM increased the digestibility of CP compared to the PC group. Ma et al [10] reported that replacing SBM with ESBM increased the apparent CP digestibility in weaned pigs. Similarly, Wang et al [20] evaluated the effect of ESBM as a partial replacement for SBM on nutrient digestibility, and the results showed that there were linear increases in coefficients of ileal apparent digestibility of DM, CP, energy, Ca, and P with increased ESBM feeding in broilers. An increase in nutrient digestion may be due to a decrease in the antinutritional factors of SEM by enzymolysis and an improvement in intestinal morphology.

Lowering the CP level in broiler diets is environmentally advantageous in that it mitigates N and ammonia emissions, thus reducing N pollution. By using a meta-analysis approach, Cappalaere et al [24] found that decreasing the dietary CP level decreased N intake and excretion and increased N retention efficiency by 2% per point of CP reduction in both pigs and broilers. Similarly, in this study, we also found that reducing the dietary CP content by 1% significantly decreased N intake and excretion in broilers. It is worth noting that dietary supplementation with ESBM did not affect litter moisture but decreased N excretion in broilers. As observed by Wang et al [20], inclusion of ESBM in the diet could increase the apparent N digestibility, leading to decreased N excretion in broilers. Additionally, degradation of antinutritional factors and improvement in intestinal health may play an important role in reducing N excretion.

Serum biochemical parameters can reflect the metabolism status of the body to a certain extent. We did not observe a significant effect of ESBM on the concentration of serum total protein, albumin, or total cholesterol in the broilers. BUN is the product of serum total protein and protein metabolites, which reflects the dynamics of protein metabolism. Yang et al [25] fed lactating sows supplemented with enzymolytic soybean protein and showed that the serum total protein level was increased and the concentrations of BUN and triglycerides were reduced, and supplementation with enzymolytic soybean protein improved the production performance of lactating sows by regulating protein and fat metabolism. Similarly, our results showed that the ESBM-supplemented groups had a trend of linearly decreased serum triglycerides and linearly increased calcium and BUN in broilers. The linear reduction in triglycerides and increase in BUN may suggest a more efficient use of the ingested nutrients and closer to the ideal balance between the energy and protein [26], as observed in this study that dietary ESBM supplementation increased CP digestibility and decreased N excretion in broilers. Zhang et al [27] reported that reducing dietary CP by 1% and adding 1.0% to 1.5% enzymolytic protein could increase the content of serum calcium and improve dietary calcium utilization in piglets, which is consistent with the results in the present study. The reason may be that ESBM supplementation improved the intestinal morphology and expression of genes encoding mineral transporters, thus facilitating calcium transport and absorption.

Intestinal morphology is used as a marker to estimate the digestion and absorption capacity of the gut, as well as intestinal health [28]. The intestinal VH and CD correlated with the number and maturation of intestinal villus epithelial cells [29]. Shen et al [21] fed 0.3%, 0.6%, and 0.9% ESBM to broilers and found that different levels of ESBM significantly affected jejunal CD and V/C, and the addition level had a quadratic curve relationship with CD, but ESBM had no effect on the duodenal morphology on d 21 and d 42. Liu et al [30] reported that supplementation with 5 g/kg ESBM increased the villous height and V/C of the duodenum and jejunum in laying hens. Ma et al [13] also concluded that compared with NC, ESBM increased VH in the duodenum and the VH to CD ratio in the duodenum and ileum in weaned pigs. Consistent with these results, in this study, we found that ESBM increased the V/C and reduced the CD of the jejunum and duodenum in broilers. These effects might be attributed to the breakdown of antinutritional factors such as glycinin and β-conglycinin in SBM, which were demonstrated to decrease VH, increase CD and reduce the VH to CD ratio, thus having negative effects on the intestinal structure and impairing nutrient digestion and assimilating [31–33]. Another explanation may be that compared to SBM, ESBM had a higher level of small peptides after an *in vitro* enzymolytic hydrolysis and fermentation. Several studies have been demonstrated that addition of bioactive small peptides in diets can enhance intestinal VH and surface area, thus improving the morphological status of the intestine [34,35]. In this study, an improved intestinal morphology implied that ESBM may alleviate intestinal stress by maintaining or improving small intestinal morphology, thus enhancing absorption ability [10]. Collectively, dietary supplementation with ESBM increased the height and the ratio of the villus to the crypt of the duodenum and jejunum, which is conducive to growth and nutrient digestion, thus leading to deceased N excretion in broilers.

## CONCLUSION

The current study indicated that supplementation with ESBM in low-CP diets had no negative effect on growth performance but increased CP digestion, decreased N excretion and improved the morphology of the duodenum and jejunum in broilers, implying a potential substitute for SBM to formulate low-CP diets in broilers.

## Figures and Tables

**Table 1:** Analyzed nutrient composition of SBM and ESBM (air dried basis, %)

Nutrients	Content (%)	

	SBM	ESBM
Gross energy (MJ/kg)	17.37	18.17
Dry matter	87.36	89.97
Crude protein	44.12	46.40
Indispensable amino acids		
Arginine	3.11	3.17
Histidine	1.05	1.05
Isoleucine	1.74	2.00
Leucine	3.04	3.44
Lysine	2.45	2.55
Methionine	0.40	0.46
Phenylalanine	2.23	2.37
Threonine	1.65	1.67
Tryptophan	0.47	0.67
Valine	2.13	2.21
Crude ash	8.04	7.96
Ca	2.48	2.43
P	0.29	0.27
Dispensable amino acids		
Alanine	1.87	1.98
Asparticacid	5.02	4.99
Cystine	0.73	0.75
Glutamicacid	7.86	8.05
Serine	2.03	2.05
Tyrosine	1.37	1.40
Glycine	1.90	1.83
Proline	4.85	4.71

SBM, soybean meal; ESBM, enzymolytic soybean meal; Ca, calcium; P, phosphorus.

**Table 2:** Composition and nutrient levels of experimental diets (air dried basis)

Items	Starter^1)^ (d 1 to 21)		Finisher^1)^ (d 22 to 42)	
	
	PC	NC	PC	NC
Ingredients (as-fed basis, %)				
Corn	51.53	53.86	53.47	56.94
Soybean meal	29.50	27.99	10.10	7.40
Rice bran	5.00	5.00	7.00	7.00
Corn DDGS	4.00	4.00	5.00	5.00
Fish meal	3.50	2.50	3.00	2.50
Extruded soy power	0.00	0.00	16.30	16.30
Soybean oil	3.10	3.00	2.15	1.75
Limestone	1.00	1.00	0.80	0.80
CaHPO_4_	1.00	1.15	1.00	1.00
Premix^2)^	0.15	0.15	0.15	0.15
NaCl	0.17	0.17	0.25	0.25
Choline chloride	0.12	0.12	0.05	0.05
*L*-Lysine	0.34	0.42	0.26	0.34
*DL*-Methionine	0.33	0.35	0.28	0.29
*L*-Threonine	0.26	0.29	0.19	0.23
Total	100.00	100.00	100.00	100.00
Nutrient levels^3)^				
ME (MJ/kg)	12.78	12.80	13.38	13.39
Crude protein (%)	21.50	20.53	19.50	18.52
Ca (%)	0.89	0.89	0.87	0.88
Total phosphorus (%)	0.52	0.49	0.53	0.52
Lysine (%)	1.48	1.48	1.27	1.27
Methionine (%)	0.68	0.68	0.61	0.61
Threonine (%)	1.07	1.05	0.92	0.92

DDGS, dry distillers grains with solubles; ME, metabolizable energy.

1) PC, positive control; NC, negative control.

2) Provided per kg diet: vitamin A 10,000 IU, vitamin D3 1,000 IU, vitamin E 25 IU, vitamin K 0.5 mg, vitamin B_1_ 2 mg, vitamin B_2_ 8.0 mg, vitamin B_6_ 4.5 mg, vitamin B_12_ 0.01 mg, biotin 0.25 mg, folic acid 0.5 mg, pantothenic acid 10.mg, Cu (CuSO_4_·5H_2_O) 25 mg, I (KI) 10 mg, Fe (FeSO_4_·7H_2_O) 100 mg, Mn (MnSO_4_·H_2_O) 120 mg, Se (NaSeO_3_) 0.15 mg, Zn (Zn0) 80 mg.

3) Nutrient levels were calculated values.

**Table 3:** Effects of low-protein diets supplemented with enzymolytic soybean meal on growth performance of broilers

Items	PC^1)^	NC^1)^	ESBM^1)^ (%)				SEM	p-value^2)^		
	
			0.5	1.0	1.5	2.0		Trt	Lin	Qua
Daily weight gain (g/bird/d)										
d 1–21	37.6	36.1	36.2	36.8	37.2	37.9	0.32	0.549	0.067	0.758
d 22–42	68.1^a^	62.9^b^	62.9^b^	63.0^b^	63.6^b^	64.7^b^	1.56	0.023	0.418	0.103
d 1–42	52.8^a^	49.4^b^	49.5^b^	49.4^b^	50.3^b^	51.0^ab^	0.99	0.012	0.088	0.397
Daily feed intake (g/bird/d)										
d 1–21	50.9	53.1	50.3	50.4	51.0	49.3	1.89	0.812	0.264	0.698
d 22–42	124	119	119	119	120	121	1.61	0.199	0.413	0.791
d 1–42	87.5	85.8	84.4	84.5	85.3	86.3	2.50	0.559	0.582	0.321
Feed conversion rate (g:g)										
d 1–21	1.36	1.47	1.39	1.38	1.38	1.32	0.05	0.331	0.038	0.725
d 22–42	1.82	1.89	1.88	1.86	1.84	1.83	0.05	0.295	0.034	0.931
d 1–42	1.60	1.68	1.67	1.64	1.61	1.58	0.05	0.168	0.012	0.752

SEM, standard error of mean.

1) PC, positive control; NC, negative control; ESBM, enzymolytic soybean meal.

2) Trt, treatment; Lin, linear; Qua, quadratic.

a,b Means in a row without a common superscript letter differ significantly at p<0.05, which were comparisons between the treatment based on a multivariate analysis of variance.

**Table 4:** Effects of low-protein diets supplemented with enzymolytic soybean meal on apparent digestibility of nutrients of broilers during fecal collection (%)

Items	PC^1)^	NC^1)^	ESBM^1)^ (%)				SEM	p-value^2)^		
	
			0.5	1.0	1.5	2.0		Trt	Lin	Qua
Dry matter	0.85	0.86	0.86	0.86	0.87	0.86	0.48	0.350	0.139	0.242
Crude protein	0.62^b^	0.64^ab^	0.67^a^	0.67^a^	0.65^ab^	0.64^ab^	0.01	0.038	0.362	0.056
Ether extract	0.72^b^	0.74^b^	0.75^b^	0.82^a^	0.75^b^	0.76^b^	0.02	0.029	0.550	0.082
Calcium	0.46	0.46	0.47	0.47	0.47	0.47	0.02	0.093	0.145	0.618
Phosphorus	0.39	0.40	0.40	0.40	0.41	0.40	0.03	0.815	0.205	0.564
Energy	0.73	0.75	0.76	0.76	0.76	0.77	0.01	0.315	0.256	0.383

SEM, standard error of mean.

1) PC, positive control; NC, negative control; ESBM, enzymolytic soybean meal.

2) Trt, treatment; Lin, linear; Qua, quadratic.

a,b Means in a row without a common superscript letter differ significantly at p<0.05, which were comparisons between the treatment based on a multivariate analysis of variance.

**Table 5:** Effects of low-protein diets supplemented with enzymolytic soybean meal on intake and digestibility of N, excreta composition and N excretion in broilers

Items	PC^1)^	NC^1)^	ESBM^1)^ (%)				SEM	p-value^2)^		
	
			0.5	1.0	1.5	2.0		Trt	Lin	Qua
N										
Intake (g/d)	4.90^a^	4.43^b^	4.61^b^	4.37^b^	4.49^b^	4.47^b^	0.40	0.011	0.114	0.632
Digested (g/d)	3.04^a^	2.85^b^	3.08^a^	2.92^ab^	2.95^b^	2.90^b^	0.22	0.025	0.121	0.226
Digestibility (%)	0.62^b^	0.64^ab^	0.67^a^	0.67^a^	0.66^a^	0.65^ab^	0.12	0.038	0.362	0.056
Excreta										
Moisture (%)	78.22	77.21	76.57	76.27	77.38	76.72	0.75	0.157	0.921	0.524
N (%)	4.70^a^	4.38^b^	4.41^b^	4.44^b^	4.36^b^	4.37^b^	0.06	0.001	0.009	0.194
N (g/kg of diet)	11.85^a^	10.67^ab^	10.36^ab^	9.79^b^	10.04^b^	9.76^b^	0.86	0.020	0.007	0.317

SEM, standard error of mean.

1) PC, positive control; NC, negative control; ESBM, enzymolytic soybean meal.

2) Trt, treatment; Lin, linear; Qua, quadratic.

a,b Means in a row without a common superscript letter differ significantly at p<0.05, which were comparisons between the treatment based on a multivariate analysis of variance.

**Table 6:** Effects of low-protein diets supplemented with enzymolytic soybean meal on serum biochemical indices of broilers

Items	Day	PC^1)^	NC^1)^	ESBM^1)^ (%)				SEM	p-value^2)^		
	
				0.5	1.0	1.5	2.0		Trt	Lin	Qua
Total protein (g/L)	21	66.15	67.06	68.27	68.46	68.63	68.98	2.02	0.999	0.672	0.890
	42	62.29	62.86	63.44	64.21	62.02	64.42	0.58	0.875	0.973	0.570
Albumin (g/L)	21	20.94	22.51	22.80	21.75	22.99	23.27	0.48	0.762	0.247	0.812
	42	20.37	20.97	20.66	20.64	21.57	21.98	0.36	0.802	0.209	0.630
Total cholesterol (mmol/L)	21	5.99	5.88	6.12	6.48	6.01	6.26	0.15	0.929	0.550	0.764
	42	11.79	11.99	11.65	12.08	11.29	12.01	0.16	0.748	0.871	0.824
Triglyceride (mmol/L)	21	6.56	6.11	6.13	6.19	6.94	6.39	0.12	0.342	0.462	0.456
	42	7.69	7.51	7.10	6.83	7.35	6.99	0.12	0.287	0.083	0.282
Calcium (mmol/L)	21	2.90	2.88	2.78	2.94	2.87	3.01	0.21	0.948	0.709	0.901
	42	2.34	2.74	2.30	2.61	2.71	2.75	0.13	0.111	0.082	0.808
Urea nitrogen (mmol/L)	21	0.17	0.19	0.18	0.17	0.17	0.18	0.01	0.970	0.986	0.880
	42	0.18	0.23	0.19	0.21	0.22	0.24	0.02	0.175	0.077	0.562

SEM, standard error of mean.

1) PC, positive control; NC, negative control; ESBM, enzymolytic soybean meal.

2) Trt, treatment; Lin, linear; Qua, quadratic.

**Table 7:** Effects of low-protein diets supplemented with enzymolytic soybean meal on duodenum jejunum mucosal morphology of broilers

Items		PC^1)^	NC^1)^	ESBM^1)^ (%)				SEM	p-value^2)^		
	
				0.5	1.0	1.5	2.0		Trt	Lin	Qua
Duodenum											
d 21	VH	1,485	1,326	1,284	1,334	1,338	1,422	71	0.077	0.132	0.283
	CD	203^a^	185^ab^	176^bc^	178^bc^	165^bc^	162^c^	10	0.003	0.023	0.948
	V/C	7.29^c^	7.20^c^	7.28^c^	7.58^c^	8.13^b^	8.76^a^	0.26	<0.001	<0.001	0.057
d 42	VH	1,985	1,381	1,477	1,398	1,414	1,565	79	0.109	0.137	0.390
	CD	214^a^	199^ab^	201^ab^	186^bc^	176^c^	183^bc^	9.8	0.005	0.013	0.596
	V/C	7.40^cd^	6.95^d^	7.36^cd^	7.52^c^	8.01^b^	8.56^a^	0.19	<0.001	<0.001	0.364
Jejunum											
d 21	VH	1,366	1,446	1,415	1,332	1,367	1,426	82	0.727	0.640	0.183
	CD	176	197	186	176	168	167	10	0.089	0.002	0.444
	V/C	7.77^bc^	7.34^c^	7.60^bc^	7.63^bc^	8.17^ab^	8.58^a^	0.28	0.002	<0.001	0.283
d 42	VH	1,191^c^	1,266^bc^	1,394^ab^	1,476^a^	1,264^bc^	1,472^a^	79	0.002	0.057	0.007
	CD	162^c^	181^ab^	197^a^	192^a^	158^c^	171^bc^	9	<0.001	0.006	0.115
	V/C	7.33^cd^	7.02^d^	7.07^d^	7.68^bc^	7.97^b^	8.59^a^	0.19	<0.001	<0.001	0.092

SEM, standard error of mean; VH, villus height; CD, crypt depth; V/C, villus height: crypt depth.

1) PC, positive control; NC, negative control; ESBM, enzymolytic soybean meal.

2) Trt, treatment; Lin, linear; Qua, quadratic.

a–d Means in a row without a common superscript letter differ significantly at p<0.05, which were comparisons between the treatment based on a multivariate analysis of variance; unit, μm.
